# Caloric reductions needed to achieve obesity goals in Mexico for 2030 and 2040: A modeling study

**DOI:** 10.1371/journal.pmed.1004248

**Published:** 2023-06-26

**Authors:** Francisco Reyes-Sánchez, Ana Basto-Abreu, Rossana Torres-Álvarez, Martha Carnalla-Cortés, Alan Reyes-García, Boyd Swinburn, Rafael Meza, Juan A. Rivera, Barry Popkin, Tonatiuh Barientos-Gutiérrez

**Affiliations:** 1 National Institute of Public Health, Population Health Research Center, Cuernavaca, Mexico; 2 Department of Epidemiology, University of Michigan–Ann Arbor, Michigan, United States of America; 3 School of Population Health, University of Auckland, Auckland, New Zealand; 4 GLOBE (Global Obesity Centre), Deakin University, Melbourne, Victoria, Australia; 5 Department of Nutrition at the Gillings School of Global Public Health, University of North Carolina at Chapel Hill, Chapel Hill, North Carolina, United States of America; 6 Department of Nutrition, University of North Carolina at Chapel Hill, Chapel Hill, North Carolina, United States of America; University of Cambridge, UNITED KINGDOM

## Abstract

**Background:**

In Mexico, obesity prevalence among adults increased from 23% in 2000 to 36% in 2018, approximately. Mexico has not defined short- or long-term obesity goals, obscuring the level of effort required to achieve a relevant impact. We aimed to explore potential obesity goals for 2030 and 2040 in Mexico and to estimate the required caloric reductions to achieve them.

**Methods and findings:**

We obtained anthropometric and demographic information on the Mexican adult population (age ≥20 years) from the Health and Nutrition Surveys conducted in 2000, 2006, 2012, 2016, and 2018 (*n* = 137,907). Each survey wave is cross-sectional, multistage, and representative of the Mexican population at the national, regional, and urban/rural levels. Obesity prevalence was projected for 2030 and 2040 by combining population projections of energy intake by socioeconomic status (SES) with a weight-change microsimulation model taking into account individual-level information on sex, age, physical activity, and initial body weight and height. If current trends continue, Mexico’s obesity prevalence is expected to increase from 36% (95% CI 35% to 37%) in 2018 to 45% (uncertainty interval [UI] 41% to 48%) in 2030 and to 48% (UI 41% to 55%) in 2040. Based on expert opinion, we identified 3 obesity goals scenarios: (1) plausible (38% in 2030 and 36% in 2040); (2) intermediate (33% in 2030 and 29% in 2040); and (3) ideal based on the average prevalence of Organization for Economic Co-operation and Development countries (OECD; 19%). We estimated the caloric reductions needed to achieve the goal scenarios using the microsimulation model. Obesity was projected to increase more rapidly in the low SES (around 34% in 2018 to 48% (UI 41% to 55%) in 2040), than in the middle (around 38% to 52% (UI 45% to 56%)), or high SES group (around 36% to 45% (UI 36% to 54%)). Caloric reductions of 40 (UI 13 to 60), 75 (UI 49 to 95), and 190 (UI 163 to 215) kcal/person/day would be needed to reach the plausible, intermediate, and the ideal (OECD) average scenarios for 2030, respectively. To reach the 2040 goals, caloric reductions of 74 (UI 28 to 114), 124 (UI 78 to 169), and 209 (UI 163 to 254) kcal/person/day would be required, respectively. Study limitations include assuming a constant and sedentary physical activity level, not considering cohort-specific differences that could occur in the future, and assuming the same caloric trends under no intervention and the obesity goal scenarios.

**Conclusions:**

To reach the 3 obesity goals in 2040, caloric reductions between 74 and 209 kcal/day/person would be needed in Mexico. A package of new and stronger interventions should be added to existing efforts such as food taxes and warning labels on non-nutritious food.

## Abstract 2

Tonatiuh Barrientos-Gutiérrez and co-workers model anticipated changes in calories consumed per person needed to achieve population obesity goals in Mexico.

## Author summary

**Why was this study done?:**

- Mexico has one of the highest obesity prevalences worldwide.
- Efforts have been made to improve dietary habits, such as implementing taxes and warning labels on non-nutritious food and beverages. However, obesity prevalence in Mexico is still increasing.
- Mexico has not defined obesity goals to help track country-level advances and assess the effectiveness of public health policies.

**What did the researchers do and find?:**

- We projected the obesity prevalence in Mexico to 2030 and 2040. These predictions were estimated using a microsimulation model of body weight, informed by projections of energy intake by socioeconomic.
- We defined 3 obesity goals based on expert opinion and the average obesity prevalence from the Organization for Economic Co-operation and Development countries (OECD): plausible (38% in 2030 and 36% in 2040), intermediate (33% in 2030 and 29% in 2040), and ideal (19%). We estimated the caloric reductions needed to attain these goals.
- Obesity prevalence is expected to increase from 36% (95% CI 35% to 37%) in 2018 to 48% (uncertainty interval [UI] 41% to 55%) in 2040. To reach the obesity goals for 2040, caloric reductions of 74 kcal/person/day (UI 28 to 114), 124 kcal/person/day (UI 78 to 169), and 209 kcal/person/day (UI 163 to 254) would be needed for the plausible, intermediate, and ideal scenarios, respectively. These goals would require higher caloric reductions for people of low socioeconomic position than people of other socioeconomic levels.

**What do these findings mean?:**

- Defining obesity goals and estimating the required caloric reductions to achieve them are needed to assess the required impact of interventions and attain meaningful population health advances.
- The obesity challenge in Mexico is substantial and requires a comprehensive package of interventions to achieve obesity goals, addressing the mechanisms affecting poor diets and weight gain.

## Introduction

The nutritional transition, characterized by the shift from traditional diets to modern energy-dense diets composed mainly of ultra-processed food, has fueled the obesity epidemic in Mexico [1]. From 2000 to 2018, the obesity prevalence increased from around 30% to 39% among women and from about 20% to 31% among men [2]. Efforts are currently underway to slow down the increasing obesity trend through structural interventions, such as taxes and front-of-package warning labels [3,4]. Still, to date, no country has stopped the increase in obesity or reversed its trend [5,6].

Over the last decades, health goals have been set to guide policy decision-making. The 66th World Health Assembly in 2013 established goals of no increase in diabetes and obesity prevalence [7], a goal to which Chile and Brazil officially committed [8,9], while the US proposed a 10% obesity reduction for 2030 [10]. Setting obesity goals is complex, as they need to consider obesity trends, prevalence, and distribution, as well as past and future interventions, combined with the ability of a society to implement them [11]. Establishing obesity goals for Mexico is urgent, given the obesity-increasing trends, the considerable current and projected burden of cardiometabolic disease, and the policies implemented and planned to be implemented.

Previous studies have constructed mathematical models to estimate changes in energy intake to meet obesity goals [12–14]. In the United States, reductions of approximately 64 kcal/day and 166 kcal/day for children and adults, respectively, would be necessary to achieve the Healthy People 2020 goals over 10 years, keeping physical activity and energy trends constant [12,13]. In low- and middle-income country (LMIC), we could not find a mathematical model to predict caloric changes to achieve obesity goals. We aimed to project the obesity prevalence in Mexico for 2030 and 2040, explore potential obesity goals, and estimate the caloric reductions required to attain them. We developed a model that includes energy projections using cross-sectional data and a microsimulation model to predict obesity. We designed a questionnaire for experts to establish plausible and intermediate obesity goals. Applying the model, we estimated the caloric intake reductions needed to reach the experts’ obesity prevalence goals.

## Methods

### Data sources

We used anthropometric and sociodemographic information of the adult population (age ≥20 years) from the Mexican Health and Nutrition Surveys (ENSANUT) conducted in 2000, 2006, 2012, 2016, and 2018. Each ENSANUT is a cross-sectional, multistage, stratified, and cluster-sample survey representative of national, regional, rural, and urban populations. We did not include ENSANUT 2020 or 2021 in the trends, as have smaller sample sizes since they are designed to produce moving average estimates after 5 years and could also include externalities related to the Coronavirus Disease 2019 (COVID-19) pandemic. The design and methods of each survey were described elsewhere [15–19]. Informed consent was obtained from each participant. At each wave, the survey research protocol was approved by the ethics, biosafety, and research committees of the Mexican National Institute of Public Health. This study is reported as per the Strengthening the Reporting of Observational Studies in Epidemiology (STROBE) guideline (S1 STROBE Checklist).

### Anthropometric information

Body weight and height were measured following standardized procedures across survey waves [20]. We calculated the body mass index (BMI) using the standard equation and categorized it into normal, overweight, and obese based on the WHO classification [21]. We included men and non-pregnant and non-lactating women with complete anthropometric information and aged ≥20 years (*n* = 139,369). We excluded extreme values for height (<130 cm or >200 cm; *n* = 667) and BMI (<10 or >59 kg/m2; *n* = 795). The analytical sample included 5 ENSANUT waves, with a total of 137,907 adults. Each wave was expanded using wave-specific survey weights.

### Socioeconomic status indicator

The indicator of socioeconomic status (SES) has changed over time across waves of ENSANUT. Thus, we constructed a standardized definition of socioeconomic status for all waves following the methodology from the ENSANUT 2018 [22]. We conducted a principal components analysis (PCA) with polychoric correlation matrix [23] using the following household characteristics: number of rooms, amenities (running water, toilet and sewage system, kitchen as a separate room, and type of fuel used for cooking), construction materials (wall, roof, and floor), and appliances (refrigerator, washing machine, microwave, stove, water heater, television, cable, radio, telephone, and computer). We considered the first component, accounting for 60%, 50%, 51%, 46%, and 50% of the total variability for 2000, 2006, 2012, 2016, and 2018 surveys, respectively. For each survey, the indicator was then classified into 3 categories (low, medium, and high) using the tertiles of the weighted index distribution as cut-off points.

### Microsimulation approach

**Fig 1:**
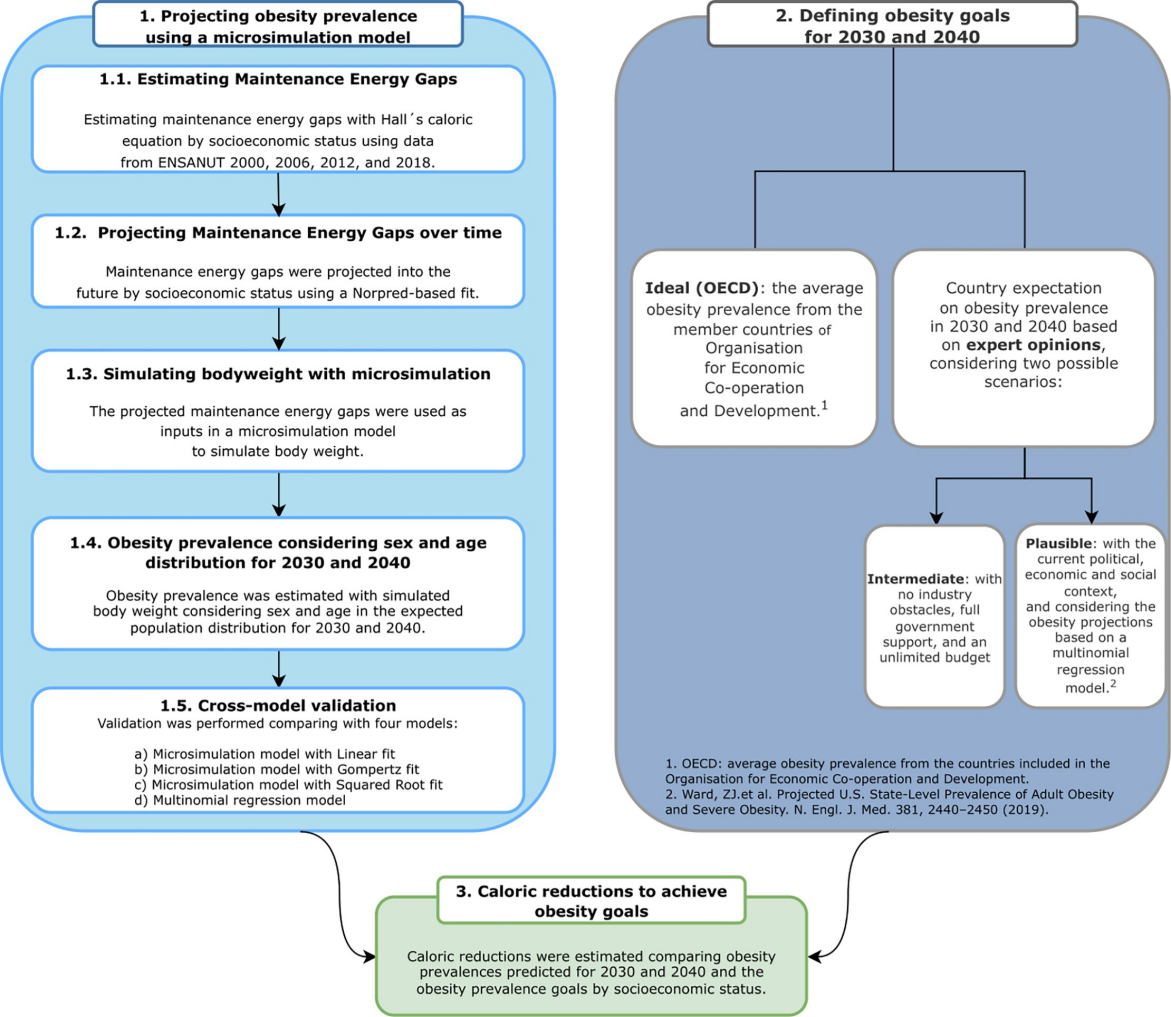
Framework summarizing the steps to model caloric gaps for 2030 and 2040.

We simulated body weight using a deterministic and dynamic microsimulation model developed by Hall and colleagues [24]. Briefly, the model estimates the expected body weight as the sum of extracellular fluid, glycogen, fat mass, and lean tissue, dependent on sodium and energy intake, energy expenditure, and individual characteristics such as sex, age, and physical activity level. This model has been previously used in Mexico assuming that under no caloric intervention, body weight remains constant over time (steady-state assumption) [25]. In this project, we removed the steady-state assumption in the model by including current population positive trends in energy intake using maintenance energy gaps (MEGs) by SES. MEGs are defined as the increase in average energy intake needed to maintain a higher body weight compared with an initial time *t*_0_ [24,26]:

$$MEG(T)=I(T)-I(t_{0})>0,$$ (1)

where *I*(*T*) and *I*(*t*_0_) are the average energy intake for time *T* and *t*_0_ (*T*>*t*_0_), respectively. MEGs were estimated by SES using previous ENSANUT waves, then projected to 2030 and 2040, and used as inputs in the microsimulation model to simulate body weight over time. Fig 1 presents a summary of the modeling steps followed, detailed in the following subsections.

### 1. Projecting obesity prevalence using a microsimulation model

We projected the obesity prevalence of the Mexican adult population by 2030 and 2040 under no intervention (business as usual*)*. To do so, we defined the ENSANUT 2018 as the baseline sample for simulation and conducted 4 main steps: (1) estimated MEGs by SES between ENSANUT 2000 and each subsequent ENSANUT wave (2006, 2012, 2016, and 2018); (2) projected the MEGs by SES over time; (3) simulated body weights with microsimulation; (4) estimated obesity prevalence considering sex and age distribution for 2030 and 2040; and (5) cross-model validation. Each step of the projection process is detailed in the following subsections.

1.1. *Estimating maintenance energy gaps*

We estimated MEGs between the ENSANUT 2000 (*T*_0_) and each subsequent ENSANUT wave (*T*_1_ = 2006, *T*_2_ = 2012, *T*_3_ = 2016, and *T*_4_ = 2018) using an equation derived by Hall and colleagues in 2009 [27],

$$MEG(T_{n})\approx \frac{d\bar{BW}}{dt}(T_{n})\times \phi_{1}kcal/day+(\bar{BW}(T_{n})-\bar{BW}(T_{0}))\times \phi_{2}kcal/kg/d,$$ (2)

where $\bar{BW}(T_{n})$ is the average body weight at time *T*_*n*_, with *n* = 1, 2, 3, or 4. Following the methodology of Hall and colleagues (2009), we estimated the coefficients *ϕ*_1_ and *ϕ*_2_ by SES, using ENSANUT data (section 2.1 in S1 Appendix). The estimated *ϕ* coefficients and MEGs by SES are presented in Tables C and D in S1 Appendix, respectively. Hall’s equation estimates average changes in energy intake from an initial time: $\bar{\Delta I}(T)=I(T)-I(T_{0})$. In contrast to the MEG definition, $\bar{\Delta I}(T)$ can also be negative or zero due to reductions or maintenance in body weight over time. In our case, $\bar{\Delta I}(T_{n})=MEG(T_{n})>0$ due to increments of body weight from 2000 to 2018 (Fig A in S1 Appendix).

1.2. *Projecting maintenance energy gaps over time*

For projecting the MEGs into the future, our first approach was to consider linear trends. These trends fitted well to the point estimates (adjusted R-squares >0.91; Table K in S1 Appendix); however, they could overestimate results due to the large simulation period (from 2018 to 2040). To prevent that situation, we considered slope reductions assuming that energy intake would continue to increase, but at a slower pace as susceptible people to the obesogenic environment are depleted. The slope reductions were taken from a cancer incidence model, called Nordpred [28,29]: a 25% reduction in 2023 to 2027 (5 years after baseline 2018), a 50% during 2028 to 2032, and a 75% in the subsequent years (Fig C in S1 Appendix). Nordpred is a mathematical model designed to improve long-term predictions, assuming the slope will eventually flatten. The slope reductions of Nordpred can be considered arbitrary for projecting energy since they are not evidence-based or previously validated. As a form of validation, we compared the energy fit of the Nordpred-based model against 3 alternative fits: linear fit, root square fit, and a Gompertz model (Fig E in S1 Appendix). In a final validation step, we estimated obesity using each energy fit and compared results against a multinomial model proposed by Ward and colleagues [30]. As shown in Fig F in S1 Appendix, the uncertainty intervals of the Nordpred obesity predictions included the point estimates of all alternative models, except for the obesity estimated using linear energy trends by 2040. However, the Nordpred- and linear-based obesity uncertainty intervals overlapped. More details about the validation are presented in section 1.4 and S1 Appendix section 2.5. Uncertainty intervals are described in section 4 in S1 Appendix. We translated the MEG projections from *t*_0_ = 2000 to $t_{0}^{\prime }$ = 2018 to match the year of the baseline sample (ENSANUT 2018):

$${{\hat{MEG}}_{j}}^{2018}(T)={\hat{MEG}}_{j}(T)-{\hat{MEG}}_{j}(2018),$$ (3)

where ${\hat{MEG}}_{j}(T)$ is the projected MEG at time *T* for the SES group *j*. With the translation, we obtained MEGs with an initial time of $t_{0}^{\prime }$ = 2018, which was needed to simulate body weights in the next step.

### 2. Simulating body weight with microsimulation

We simulated body weight from the baseline sample (ENSANUT 2018) using Hall’s microsimulation model and the projected MEGs. First, we assigned a MEG projection $\vec{{MEG}_{k}}(t)$ to each individual *k* in the baseline sample, according to its SES group. Then, body weight was simulated for each individual *k* in the baseline sample as [31]:

$$BW_{k}(t)=BW_{k}^{model}\left(age_{k}+t;Sex_{k},Height_{k}(0),BW_{k}(0),EIchange=\vec{{MEG}_{k}}(t),\vec{PAL_{k}}(t),\vec{\Delta NA_{k}}(t)\right),$$ (4)

where *Height*^*k*^(0) and *BW*_*k*_(0) denote the initial height and body weight, respectively. *EIchange* represents daily changes in energy intake from the baseline year ($t_{0}^{\prime }$ = 2018). In our analyses, *EIchange* corresponded to the MEG projection assigned to each individual in the baseline sample (ENSANUT 2018). ${\overset{⃑}{PAL}}_{k}$ denotes daily physical activity levels, and $\vec{\Delta NA_{k}}(t)$ represents the daily changes in sodium, accounting for changes in extracellular fluids considered in the model. We assumed a sedentary level of physical activity, which was kept constant over time (${\overset{⃑}{PAL}}_{k}=\overset{⃑}{1.5}$), and no changes in sodium intake (${\overset{⃑}{\Delta NA}}_{k}=\overset{⃑}{0}$). We decided to keep sedentary levels constant over time as a conservative scenario for energy change, considering that sedentarism has been increasing over time in Mexico [32,33]. Under those assumptions, the model estimated changes in body weight triggered by the projected MEGs. In previous articles using Hall’s model in Mexico, *EIchange* in Eq (4) had negative values only, as a result of the intervention effect [25]. In this model, *EIchange* reflects increasing trends in energy intake under a business as usual scenario, based on current intake trends. More details of the simulation process are available in S1 Appendix section 2.3.

2.1. *Obesity prevalence considering sex and age distribution for 2030 and 2040*

BMI was estimated for 2030 and 2040, using the body weight predictions, assuming constant height over time. Individuals with a BMI ≥30 kg/m^2^ were assigned to the obesity category, according to the WHO’s BMI classification. We considered expected changes in sex and age distribution for 2030 and 2040 using the projected sex and age distribution according to Mexico’s National Population Council (section 2.4 in S1 Appendix) [34]. For that, we used *raking*, a sampling balance method [35], to adjust the sampling weights to replicate the expected age and sex distribution in 10-year age groups for 2030 and 2040. This method allowed us to account for aging, migration, deaths, and births in our obesity prevalence predictions.

2.2. *Cross-model validation*

We compared our results with 4 independent models: 3 different fits integrated in the microsimulation models and 1 population-level model. Microsimulation models applied a similar model structure as in the main scenario, but to project caloric changes, we changed the Nordpred energy-intake fit to linear, Gompertz, or squared root fit. The population-level model applied a multinomial regression model following the work of Ward and colleagues, previously used to predict obesity trends in the US [30]. Specific methods for the cross-model validation are explained in section 2.5 in S1 Appendix.

### 3. Defining obesity goals for 2030 and 2040

We developed 3 scenarios for obesity reductions for 2030 and 2040: (1) plausible and (2) intermediate scenarios based on expert opinion, and (3) ideal scenario based on the obesity OECD average prevalence in 2017 (19%) [36]. We elicited expert opinion using a previously piloted questionnaire in Spanish (see section 3 in S1 Appendix for more details on the questionnaire design and piloting). The questionnaire focused on obtaining obesity prevalence goals in Mexico for 2030 and 2040, assuming 2 scenarios: (1) an intermediate scenario with no industry obstacles, full governmental support, and an unlimited budget; and (2) a plausible scenario considering the current political, economic, and social context in Mexico and the obesity projections using the multinomial regression model (explained in detail in section 2.5.4 in S1 Appendix). We constructed a multiple choice answer with ranges of obesity prevalence for both scenarios, as follows: *What obesity prevalence could be reached in 2030 and 2040 under an ideal scenario*? As a reference, the prevalence of obesity in Mexican adults in 2018 was 36% (95% CI 35% to 37%). We invited 9 obesity and nutrition experts from Mexican institutions and obtained 8 anonymous answers (88% response rate). We estimated the median of the selected obesity goals.

### 4. Caloric reductions to achieve obesity goals

To attain obesity prevalence goals, we estimated caloric reductions by SES based on the projected *MEGs* between $T_{0}^{\prime }$ = 2018 and *t*_1_ = 2030 or *t*_2_ = 2040. Applying these MEGs as reductions, we estimated the caloric increase that explains the projected increase in obesity prevalence. Then, we looked for a constant *K* energy for all SES groups such that the caloric reduction *MEG*_*j*_(*T*) + *K* would translate into the obesity prevalence goal for *T* = 2030 or *T* = 2040 using Hall’s microsimulation model. Using this methodology, we assumed the caloric reductions do not modify the energy slope over time, as it only affects the intercept between $T_{0}^{\prime }$ and *t*_1_ or *t*_2_. Fig G in S1 Appendix presents an example of the Nordpred-based projections with and without a caloric reduction for the low SES. The caloric reductions only represent geometric translations of the projections. To express the caloric gaps as percentages of the current intake, we estimated the energy intake for each individual in the baseline sample (ENSANUT 2018) using the following equation [37]:

Energyintake≈PAL×REE, (5)

where PAL is the physical activity level (assumed sedentary = 1.5) and REE is the resting energy expenditure. The REE was estimated using Mifflin’s equation [38]:

REE=9.99∙bodyweight+625∙height−4.92∙age+5∙sex−161, (6)

were sex = 1 for males and sex = 0 for females. For estimating Eq (6), we assumed that individuals were approximately in energy equilibrium by 2018 (Energy intake ≈ Energy expenditure). The estimated energy intakes by socioeconomic statutes are presented in Table M in S1 Appendix.

### 5. Uncertainty intervals

To add uncertainty in the model, we simulated 50,000 times the energy trajectories considering the variance from the body weights, estimated with the ENSANUT waves, and the variance from the Nordpred-based fit. First, we simulated 1,000 times the body weights, assuming they had a normal distribution (means and standard deviations are presented in Table A in S1 Appendix). For each of these 1,000 simulations, we estimated and projected the MEGs with the Nordpred-based fit. Then, each Nordpred-based fit was simulated 50 times using the variance of its predictions, producing 1,000 × 50 = 50,000 projections of MEGs. We took the 2.5% and the 97.5% percentiles of the simulated fitted energies as the uncertainty range around the Nordpred-based fit (Fig H in S1 Appendix). The 2.5% percentiles were used as an optimistic scenario of intake change over time, and the 97.5% percentiles as a pessimistic scenario. With these scenarios, we repeated the simulation process and estimated an interval for both projected obesity prevalences and caloric reductions. Uncertainty from the microsimulation weight-change model was not assessed due to computational time but could be implemented by simulating the model parameters [27]. Instead, we conducted the cross-model validation to consider other possible MEG projections for estimating body weight. The uncertainty methodology used for the Nordpred-based fit was also used to estimate uncertainty intervals (UIs) for the root square and the Gompertz models.

## Results

**Fig 2:**
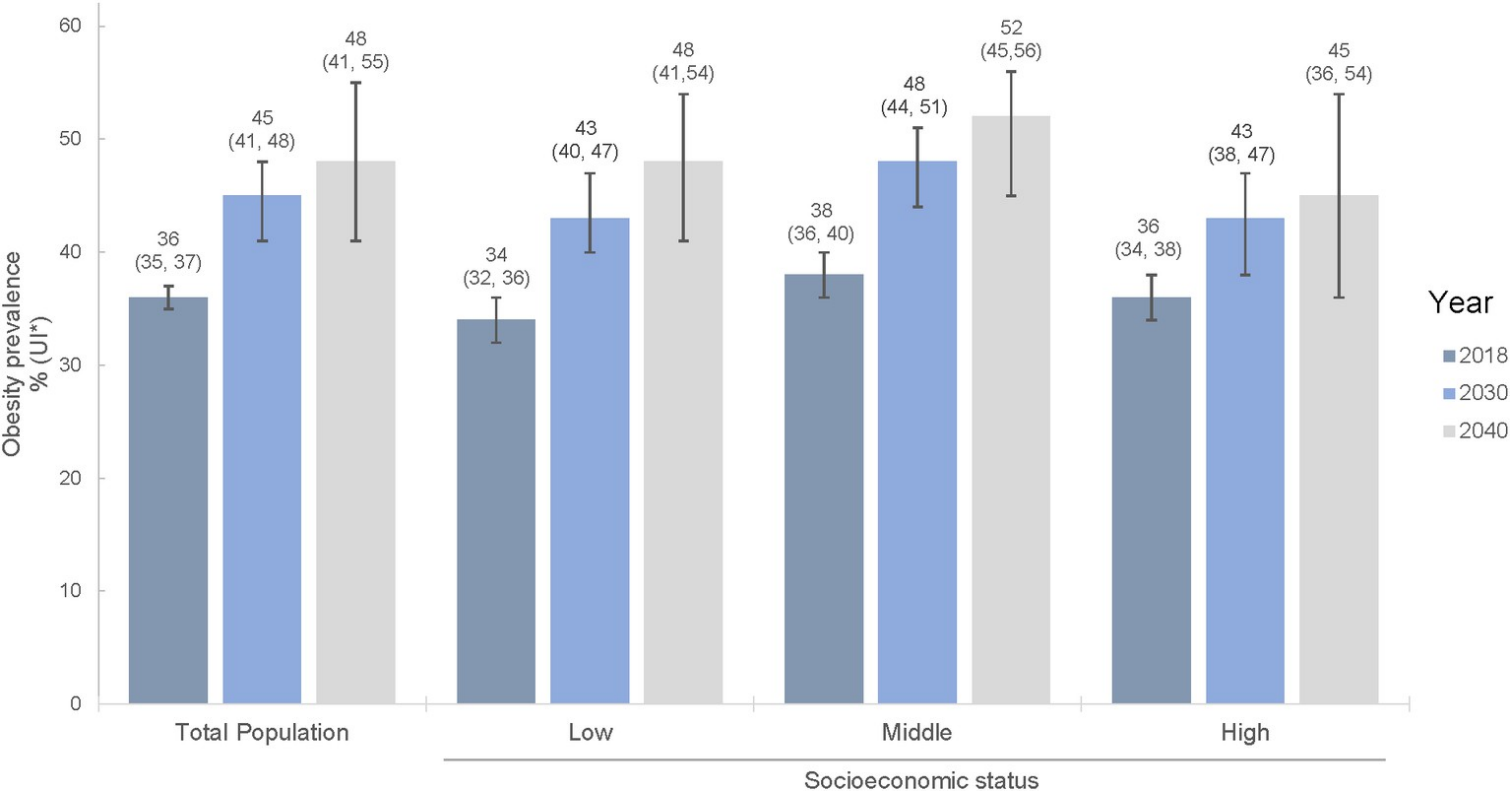
Obesity prevalence microsimulations by socioeconomic status, considering age and sex distribution in 2030 and 2040 under no intervention (business as usual). UI, uncertainty interval.

Fig 2 presents obesity projections for the overall population and by SES under no intervention (business as usual scenario). Obesity prevalence was projected to increase from the observed 36% (95% CI 35% to 37%) in 2018 to 45% (UI 41% to 48%) in 2030 and to 48% (UI 41% to 55%) in 2040. Obesity was projected to increase more rapidly in the low SES group (34% (95% CI 32% to 36%) in 2018 to 48% (UI 41% to 54%) in 2040), than in the middle (38% (95% CI 36% to 40%) to 52% (UI 45% to 56%)), or high SES group (36% (95% CI 34% to 38%) to 45% (95% CI 36% to 54%)). Still, obesity prevalence in 2040 was predicted to be the highest among middle SES (about 52%), followed by low (about 48%) and then high SES (about 45%).

Table 1 shows the 3 scenarios for obesity goals in 2030 and 2040: the ideal (OECD), and the intermediate, and the plausible scenario according to expert opinion. The OECD average obesity prevalence was 19% [36], targeted to be achieved in 2030 or 2040. Using expert opinion, the intermediate scenario aimed to achieve a 33% obesity prevalence for 2030 and 29% for 2040, the plausible scenario aimed to achieve a 38% obesity prevalence for 2030 and 36% for 2040. Table 1 also presents the decrease in caloric intake needed to meet the proposed obesity goals. According to the model, to achieve the ideal (OECD) scenario for 2030, a reduction of 190 kcal/person/day (UI 163 to 215) would be needed, which represents 9% (UI 8% to 10%) of the total energy intake (TEI) estimated at baseline 2018 (Table N in S1 Appendix). To reach the intermediate scenario for 2030, a reduction of 75 kcal/person/day (UI 49 to 95) from the baseline intake (4% of the TEI; UI 2% to 4%) would be needed, while for the plausible scenario, the reduction would be 40 kcal/person/day (2% of the TEI; UI 1% to 3%). In all cases, higher energy intake reductions would be needed in the low SES group versus the middle and high SES groups. To reach the ideal scenario for 2040, adults in Mexico would need to reduce 209 kcal/person/day (UI 163 to 254) from their initial intake (10% of the TEI; UI 8% to 12%), 124 kcal/person/day (UI 78 to 169) to achieve the intermediate scenario (6% of the TEI; UI 4% to 8%), and 74 kcal/person/day (UI 28 to 114) to achieve the plausible scenario (4% of the TEI; UI 1% to 5%).

**Table 1:** Obesity prevalence goals in 2030 and 2040 by socioeconomic status and caloric change needed to achieve them.

	Obesity prevalence goals (percentage points, pp)			Caloric gaps to achieve the goals			
				(kcal/person/day)			
Scenario	Obesity goal	Change respect to baseline (2018, pp)^a^	Change respect to projections ^b^	Socioeconomic status			
				Total population	Low	Middle	High
**2030**							
Ideal (OECD)^c^	19	−17	−26	−190 (−215, −163)	−206 (−228, −184)	−190 (−210, −168)	−175 (−207, −141)
Intermediate^d^	33	−3	−12	−75 (−95, −49)	−91 (−108, −69)	−75 (−90, −53)	−60 (−87, −27)
Plausible^e^	38	1^f^	−7	−40 (−60, −13)	−56 (−73, −34)	−40 (−55, −18)	−25 (−52, 9)
**2040**							
Ideal (OECD)	19	−17	−29	−209 (−254, −163)	−231 (−271, −190)	−210 (−245, −174)	−189 (−249, −129)
Intermediate	29	−8	−19	−124 (−169, −78)	−146 (−186, −105)	−125 (−160, −89)	−104 (−164, −44)
Plausible	36	0	−12	−74 (−114, −28)	−96 (−131, −55)	−75 (−105, −39)	−54 (−109, 6)

^a^ Obesity prevalence estimated at the baseline year (2018): 36% (95% CI 35%–37%). pp = percentage points. CI = confidence interval.

^b^ Projected obesity prevalence: 45% (UI 41%–48%) by 2030 and 48% (UI 41%–55%) by 2040.

^c^ OECD: average obesity prevalence from the countries included in the Organization for Economic Co-operation and Development in 2017.

^d^ Intermediate scenario: with no industry obstacles, full governmental support, and an unlimited budget.

^e^ Plausible scenario: considering the current political, economic, and social context under the obesity projections based on the multinomial regression model.

^f^ This change represents an increment respect to the baseline obesity prevalence but a reduction respect to the projected prevalence by 2030 (45%), which was considered by the experts to define this plausible scenario.

## Discussion

In this study, we projected the obesity prevalence in Mexico using a microsimulation model that integrates projections of MEGs at the population level and estimated the caloric reduction needed to achieve obesity goals for 2030 and 2040. We found that, under a business as usual scenario, obesity prevalence among adults is expected to increase from 36% (95% CI 35% to 37%) in 2018 to 45% (UI 41% to 48%) in 2030 and to 48% (UI 41% to 55%) in 2040. For 2040, it would be needed to reduce 209 (UI 163 to 254) kcal/person/day to reach the ideal (OECD) scenario (19% of obesity prevalence), 124 (UI 78 to 169) kcal/person/day to achieve the intermediate obesity goal (29%), and 74 (UI 28 to 114) kcal/person/day to achieve the plausible obesity goal (36%). To reach obesity goals, all adults would need to reduce significantly their energy intake, but people in the low SES group would need to reduce their calorie intake the most.

Caloric gaps to reach obesity goals have been reported in the US for the adult population [12]. For that, a stochastic microsimulation approach was used to estimate the changes in energy intake and physical activity needed to meet the Health People (HP) 2020 obesity goal (10% reduction) [12]. This goal was set by the US Federal Interagency Workgroup based on the HP 2010 results [39]. To reach the HP 2020 obesity goal, the US study estimated that a reduction of around 166 kcal/person/day (8.5% of the daily energy intake) would be needed, reducing about 6 pp the obesity prevalence in 10 years (from 36.4% to 30.5%). We estimated that 75 kcal/person/day (4.0% of the daily energy intake) would be needed to reduce approximately 3 pp the obesity prevalence (from 36% to 33%) in 12 years for reaching the intermediate scenario for 2030. Differences could be explained by different physical activity assumptions (we assumed a sedentary and constant physical activity level, while the US study predicted reductions in physical activity over time). Reducing physical activity, a higher reduction in energy intake would be needed to reach the same obesity prevalence.

Estimating energy gaps to achieve obesity goals is key to inform the magnitude of population interventions needed to attain meaningful advances. In Mexico, a 10% tax on sugar-sweetened beverages could reduce 8 kcal/person/day [40–42], and an 8% tax on energy-dense food would reduce 14 kcal/person/day [43]. Similarly, the recently implemented front-of-pack warning labels for processed foods are expected to reduce about 37 kcal/person/day [25]. In isolation, each intervention falls short of the minimum 40 kcal/person/day needed to achieve the plausible scenario for 2030, and even shorter from the about 75 kcal/person/day and 190 kcal/person/day to achieve the intermediate and ideal (OECD) scenarios. This comparison highlights the need to update all available measures, for example by increasing taxes, and to implement a comprehensive package of interventions to help reduce the caloric intake. Individual-level interventions will also be required to target specific subpopulations already experiencing obesity or at higher risk of significant weight gain [44]. Chile implemented a nationwide package of interventions that consisted of taxes and marketing restrictions linked to front-of-pack labels that produce significant reformulation and changes in consumption, leading to a reduction of approximately 49.4 kcal/person/day [45]; this model of intervention could be useful for Mexico. Other efforts for tackling the obesity epidemic are underway in Mexico, such as changes to the General Education Law on Healthy School Environments to incorporate multiple interventions targetting children: banning sales of unhealthy food and drinks in schools, prohibiting advertising, prioritizing local products in school cafeteria, and prohibiting sales in vending machines [46].

This study predicts an increase in obesity disparities. The obesity rate is predicted to increase faster among the low SES group (about a 42% increase) than the high SES group (about 25% increase) until 2040. The rapid obesity increase in the low SES is alarming and could deepen the already existing inequities in the country and perpetuate the poverty cycle, especially with its expected rapid raise in diabetes prevalence [47]. This result could be due to lower access to healthy foods and increasing intakes of ultra-processed and industrialized food in the low socioeconomic group [48,49]. To reach the obesity goals, the low SES group would need to reduce more calories than the high SES group and so, interventions with higher equitable effects are needed, such as taxation [44]. We know that a 20% tax to all processed food and beverages with warning labels would impact low SES populations the most [44]. Tax revenue could then be used to increase the availability of drinking water access and to subsidize access to healthier food options for low-income individuals (e.g., via a food stamp or similar program) [50]. While the obesity is increasing in a higher pace for low SES, the middle SES group is predicted to have the highest obesity prevalence by 2040. The interventions aforementioned would also be beneficial for the middle SES group.

Our predictions largely depend on our assumptions. As a cross-validation, we reestimated obesity projections using different energy intake fits and a multinomial regression model. The models produced comparable results (section 2.5.5 in S1 Appendix). For 2030, obesity prevalence could reach rates between 43% and 48%, using microsimulation with the root square fit and the Gompertz model, respectively, compared to 45% estimated in our main model (Fig F in S1 Appendix). For 2040, obesity could reach rates between 48% and 56%, with our main model and the linear fit, respectively. These ranges are consistent with previous obesity projections for Mexico in 2030 (Table L in S1 Appendix) [51]. In a study in the US, using a similar multinomial regression model, the authors estimated an obesity prevalence of 49% (UI 48% to 50%) for 2030 [30], compared with 48% (UI 41% to 55%) found in our study in 2040. Compared to European countries in 2025, comparable estimates were found for Ireland, 43% (UI 28 to 58) versus 45% (UI 41% to 48%) in Mexico in 2030 [52].

Several limitations need to be acknowledged. Microsimulation modeling has several advantages and limitations that have been addressed in more depth elsewhere [53]. In the microsimulation model, we assumed that physical activity would be constant over time and the only source of imbalance was the energy intake through the projected MEGs from 2018 to 2040. That assumption was taken as a conservative scenario for energy change, considering that sedentarism has been increasing over time in Mexico [32,33]. To consider age and sex changes over time into our obesity 2030 and 2040 obesity predictions, we constructed new population sample weights to approximate the sex and age distributions for the simulated years. Still, the model does not consider cohort changes, i.e., children and adolescents who by 2040 would become adults, and could be subject to generational behaviors or caloric intake patterns different from 2018 adults. For the simulation process, we are not considering any externalities into the future that might shift dramatically the trend, such as COVID-19. Although, the COVID-19 pandemic had a major impact on the population’s lifestyle, external to previous behaviors, specifically on physical activity, nutritional patterns, and food insecurity due to lockdowns [54,55]; it is hard to estimate the magnitude or the direction of this potential bias. Additional surveys post COVID would be needed to recalibrate the model. We are estimating energy trends by SES subpopulations. Trends might differ across other stratums such as age and sex; however, our main goal is to evidence SES inequities in obesity. Of note, SES provides us a relative measure of inequity as the tertiles were estimated for each survey wave. We are using BMI as an outcome, which could be a simplification of obesity as more accurate methods exist to measure body fat levels. However, BMI still represents a practical tool to estimate and compare overweight and obesity in population studies [56–58]. To simulate the obesity goals, we applied constant caloric reductions by SES from the baseline to the end of the simulation period, assuming that the caloric reductions would not affect the slope of the intake trends. Additional methodology considering the effect of caloric interventions on the energy trends needs to be considered in future studies. The estimated caloric reductions in our study are informative to evaluate efforts needed to achieve obesity goals, but the intake changes should be applied immediately given the estimated increasing trends in intake. If they are applied later, they can be insufficient to reach the obesity goals.

Obesity prevalence in Mexico has been increasing over the last 2 decades. We predicted that obesity prevalence will reach nearly 50% by 2040, with the highest increase in the low SES (about 42% increase). Models are helpful to gauge the expected impact of these interventions on future obesity trends. Our study highlights the need for countries facing significant weight increases to project future scenarios, define obesity goals, and estimate the effort needed to attain them. The model used in this study could be useful to other countries to estimate their own obesity trends and caloric changes using the same metabolic microsimulation model, but adding country-specific caloric trends and population conditions. Mexico urgently needs a comprehensive set of obesity reduction interventions targeting the food environment to decelerate the obesity epidemic and guide the population to make healthier eating choices. A package of interventions is currently being designed focusing on children and adolescents to regulate marketing, prohibit marketing of products with warning labels and ban the sale of these products in schools [50]. Also, increasing taxes to ultra-processed foods are currently being discussed [59,60].

## Supporting information

S1 STROBE Checklist Checklist of items, extended from the STROBE statement, which should be reported in observational studies using routinely collected health data. (DOCX)

S1 Appendix Additional information on the simulation model and assumptions. (PDF)

## Abbreviations

BMI: body mass index
COVID-19: Coronavirus Disease 2019
LMIC: low- and middle-income country
MEG: maintenance energy gap
OECD: Organization for Economic Co-operation and Development
PCA: principal components analysis
SES: socioeconomic status
TEI: total energy intake
UI: uncertainty interval
