# Supplementary material for: Caloric reductions needed to achieve obesity goals in Mexico for 2030 and 2040: A modeling study
Source: PLoS Med. 2023 Jun 26;20(6):e1004248. doi: 10.1371/journal.pmed.1004248 (PMC10292696; doi:10.1371/journal.pmed.1004248)
Supplement: S1 Appendix — (PDF) [file pmed.1004248.s002.pdf]

# S2 Appendix for "The caloric reduction needed to achieve the obesity goals for 2030 and 2040: a modeling study"

Francisco Reyes-Sánchez, BS[1,*], Ana Basto-Abreu, PhD[1,*], Rossana Torres-Álvarez, BS[2], Martha Carnalla-Cortés, PhD[1], Alan Reyes-García, MS[1], Boyd Swinburn, PhD[3,4], Rafael Meza, PhD[2], Juan A. Rivera, PhD[1], Barry Popkin, PhD[5,6], and Tonatiuh Barrientos-Gutiérrez, PhD [*1]

[1] *National Institute of Public Health, Population Health Research Center, Mexico*
[2] *Department of Epidemiology, University of Michigan – Ann Arbor, United States*
[3] *School of Population Health, University of Auckland, Auckland, New Zealand*
[4] *GLOBE (Global Obesity Centre), Deakin University, Melbourne, Victoria, Australia*
[5] *Department of Nutrition at the Gillings School of Global Public Health, University of North Carolina at Chapel Hill, Chapel Hill, North Carolina, United States*
[6] *Department of Nutrition, University of North Carolina at Chapel Hill, United States*

[*] *These authors contributed equally to this work*

*Contact information: Center for Population Health Research, National Institute of Public Health, Avenida Universidad 655, Santa María Ahuacatitlán, 62100 Cuernavaca, Morelos, México, [tbarrientos@insp.mx], (52)5554871015.

# Contents


**1 Data Inputs** **3**
1.1 Antropometric data . . . 3
1.2 Socioeconomic Status Indicator . . . 3
1.3 Baseline sample for microsimulation . . . 3

**2 Projecting obesity prevalence using a microsimulation model** **4**
2.1 Estimating energy maintenance gaps . . . 6
2.2 Projecting Maintenance Energy Gaps over time . . . 9
2.2.1 Maintenance energy gaps from the baseline year . . . 11
2.3 Simulating body weight using microsimulation . . . 12
2.4 Obesity prevalence considering sex and age distribution for 2030 and 2040 . . . 13
2.5 Cross-model validation . . . 16
2.5.1 Root square fit . . . 16
2.5.2 Gompertz model . . . 18
2.5.3 Comparing projections of maintenance energy gaps . . . 20
2.5.4 Multinomial regression model . . . 22
2.5.5 Comparing results for obesity prevalence . . . 22
2.5.6 Comparison with other estimations in Mexico . . . 23

**3 Defining obesity goals for 2030 and 2040** **24**
3.1 Piloting . . . 24

**4 Caloric reductions to achieve the obesity goals** **25**

**5 Uncertainty analysis** **27**

**References** **30**

# 1 Data Inputs

We used antropometric and socio-demographic data of the Mexican adult population from the National Health and Nutrition Surveys (ENSANUTs for its Spanish acronym) conducted in 2000, 2006, 2012, 2016, and 2018. Each ENSANUT is a cross-sectional, multistage, stratified and cluster-sampled survey, that is designed to quantify the frequency and distribution of health and nutrition conditions in the Mexican population, and is representative at the national, regional and area of residence level.

## 1.1 Antropometric data

Weight and height were collected by trained personnel using consistent validated and standardized methods across ENSANUT waves. For each wave, body weight was measured using digital scales with a precision of 100 g with participants wearing light clothing, and height was measured using stadiometers with a precision of 2 mm.

## 1.2 Socioeconomic Status Indicator

We constructed a socioeconomic status indicator for all ENSANUT waves using principal components analysis (PCA) with a polychoric correlation matrix [1]. We considered a number of variables within household facilities and assets: number of rooms, household amenities: running water, toilet and sewage system, kitchen as a separate room, type of fuel used for cooking, construction materials: wall, roof, and floor, and household goods and services: refrigerator, washing machine, microwave, stove, water heater, television, cable, radio, telephone, and computer. For the five survey waves, we extracted the first component (eigenvalue $> 1$), explaining 60% of the variance in 2000, 50% in 2006, 51% in 2012, 46% in 2016, and 50% in 2018.

## 1.3 Baseline sample for microsimulation

As the baseline sample for simulation, we used data corresponding to the Mexican adult population aged 20 years old from the ENSANUT 2018. After data processing, described in the main document, the baseline sample consisted of $n = 16,061$ subjects. When sampling weights were considered, those subjects expanded to $N = 75,009,850$ individuals in the adult population in Mexico. We accounted for the survey design of the ENSANUT 2018 using the "Survey" package in the R software as follows [2–4]:

```
Svy.design.2018 <- svydesign(
id = ~id, strata = ~est_var, weights = ~weights.2018,
PSU = ~code_upm, data = Adults.ENSANUT.2018)
options(survey.lonely.psu = ''adjust'')
```

# 2 Projecting obesity prevalence using a microsimulation model

We projected obesity prevalence using a microsimulation weight change model derived by Hall et al. [5]. This model estimates body weight over time taking into account the characteristics of the individuals (sex, age, height, initial body weight and physical activity), and dynamics in energy consumption, energy expenditure, and sodium intake (accounting for changes in extracellular liquids). The microsimulation model was used to simulate the body weight of the individuals in the baseline sample, considering current trends in energy consumption. These trends were estimated by socioeconomic status using the ENSANUT waves and an equation of change in intake proposed by Hall et al. 2009 [6]. The changes in intake were estimated from $T_0 = 2000$ and corresponded to increments of average body weight (Fig A, Table ), matching the *maintenance energy gap* (MEG) definition [5]. In the next subsections, we present a more detailed explanation of the steps followed to project obesity prevalence for the adult Mexican population by 2030 and 2040.

**Fig A:** Average body weight for Mexican adults aged $\geq 20$ by socieconomic status. These average values were estimated for the years 2000, 2006, 2012, 2016 and 2018 using data from the National Health and Nutrition Survey.

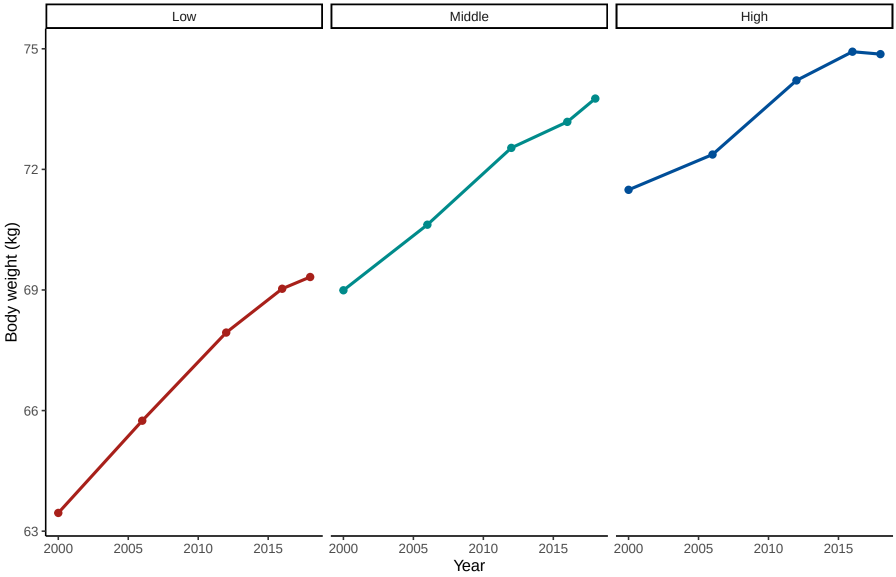

**Table A:** Average body weight for Mexican adults aged $\geq 20$ by socieconomic status. These average values were estimated for the years 2000, 2006, 2012, 2016, and 2018 using data from the National Health and Nutrition Survey.

| Socioeconomic status | Year | Average body weight | Standard deviation |
|---|---|---|---|
| Low | 2000 | 63.4 | 0.2 |
| | 2006 | 65.7 | 0.2 |
| | 2012 | 67.9 | 0.2 |
| | 2016 | 69.0 | 0.5 |
| | 2018 | 69.3 | 0.3 |
| Middle | 2000 | 69.0 | 0.2 |
| | 2006 | 70.6 | 0.2 |
| | 2012 | 72.5 | 0.2 |
| | 2016 | 73.2 | 0.5 |
| | 2018 | 73.8 | 0.3 |
| High | 2000 | 71.5 | 0.2 |
| | 2006 | 72.4 | 0.3 |
| | 2012 | 74.2 | 0.3 |
| | 2016 | 74.9 | 1.0 |
| | 2018 | 74.9 | 0.4 |

## 2.1 Estimating energy maintenance gaps

Maintenance energy gaps were estimated between $T_0 = 2000$ and $T_n = 2006$, 2012, 2016, or 2018 using the ENSANUT waves and a equation proposed by Hall et al. 2009 [6]:

$$MEG(T_n) \approx \frac{d\overline{BW}}{dt}(T_n) \times \phi_1 \text{ kcal/kg } + \left(\overline{BW}(T_n) - \overline{BW}(T_0)\right) \times \phi_2 \text{ kcal/kg/day}. \tag{1}$$

$d\overline{BW}/dt\ (T_n)$ corresponded to the rate of change in average body weight at time $T_n$ (kg/day) and was estimated by SES assuming linear trends of body weight between ENSANUT waves:

$$\frac{d\overline{BW}}{dt}(T_n) = \frac{\overline{BW}(T_n) - \overline{BW}(T_{n-1})}{(T_n - T_{n-1}) \times 365 \text{ day/year}}; \tag{2}$$

where $n = 1, 2, 3$ or 4 and $T_0 = 2000$. The coefficients $\phi_1$ and $\phi_2$ in equation (1) were estimated by SES following Hall et al. 2009 methodology:

$$\phi_1(\alpha_k) = \frac{1}{1-\beta} \times \left\{ \frac{\eta_F + \rho_F + \alpha_k \times \eta_{FFM} + \alpha_k \times \rho_{FFM}}{(1+\alpha_k)} \right\}; \tag{3}$$

$$\phi_2(\alpha_k) = \frac{1}{1-\beta} \times \left\{ \frac{\gamma_F + \alpha_k \times \gamma_{FFM}}{1+\alpha_k} + \delta \right\}. \tag{4}$$

According to Hall, $\beta = 0.24$ considers the adaptation of energy expenditure during changes in diet; $\eta_F = 180$ kcal/kg and $\eta_{FFM} = 230$ kcal/kg account for the biochemical cost of tissue deposition, assuming that the change of fat mass ($F$) and fat-free mass ($FFM$) is primarily accounted by body protein and its associated water. The coefficients $\rho_F = 9400$ kcal/kg and $\rho_{FFM} = 1800$ kcal/kg are the energy densities for changes in $F$ and $FFM$, respectively. $\gamma_F = 3.6$ kcal/kg/day and $\gamma_{FFM} = 22$ kcal/kg/day are regression coefficients explaining the relationship between the resting metabolic rate (dependant variable), and $F$ and $FFM$, respectively [7]. $\phi_1$ and $\phi_2$ in equations (3) and (4) depend only on $\alpha_k$, which stands for the relative change in average $F$ and $FFM$ for the SES group $k$. $\alpha_k$ was estimated using Forbes' equation [8]:

**Table B:** Parameters used for estimating energy gaps, proposed by Hall et al.*

| Parameter | Description | Value |
|---|---|---|
| $\beta$ | Adaptive thermogenesis | 0.24 |
| $\eta_F$ | Cost of fat synthesis | 180 kcal/kg |
| $\eta_{FFM}$ | Cost of fat free tissue synthesis | 230 kcal/kg |
| $\rho_F$ | Energy density for changes in fat mass | 9400 kcal/kg |
| $\rho_{FFM}$ | Energy density for changes in fat-free mass | 1800 kcal/kg |
| $\gamma_F$ | Metabolic rate of adipose tissue | 3.6 kcal/kg/day |
| $\gamma_{FFM}$ | Metabolic rate of fat-free tissue | 3.6 kcal/kg/day |

*Hall KD, Sacks G, Chandramohan D, Chow CC, Wang YC, Gortmaker SL, et al. Quantification of the effect of energy imbalance on bodyweight. The Lancet. 2011 8; 378(9793):826-37.

$$\alpha_k := \frac{d(\overline{FFM_k})}{d\overline{F_k}} = \frac{10.4}{\overline{F}_{0k}}; \tag{5}$$

where $\overline{F}_{0k}$ is the average fat mass for the SES group $k$ at the intial time ($T_0 = 2000$). To estimate $\overline{F}_{0k}$, we first estimated fat mass for each individual in the ENSANUT 2000, using equations derived by Jackson et al. [9]:

$$F_j = \begin{cases} \left[(4.35 \times BMI_j) - (0.05 \times BMI_j^2) - 46.24\right] \times BW_j & \text{for women} \\ \\ \left[(3.76 \times BMI_j) - (0.04 \times BMI_j^2) - 47.80\right] \times BW_j & \text{for men} \end{cases}$$

where $BMI_j$ and $BW_j$ are the body mass index and the body weight of the individual $j$ in the sample. Then, we estimated the average fat mass for each SES group in 2000 ($\overline{F}_{0k}$), considering the sampling design of the ENSANUT 2000. Table C shows the estimated values of $\overline{F}_0, \alpha$, $\phi_1$, and $\phi_2$ by SES. For the initial time $T_0$, we assumed that people's weight was in a steady-state, corresponding to a state of energy balance (energy intake = energy expenditure) [6]. In Hall's equation and microsimulation model, the energy balance assumption corresponds to no changes in energy intake at the initial time: $\overline{\Delta I}(T_0) \approx 0$ or in our case $MEG(T_0) \approx 0$.

**Table C:** Parameters used to estimate maintenance energy gaps. $\overline{F}_0$ is the average fat mass in 2000, $\phi_1$ (kcal/kg) and $\phi_2$(kcal/kg/d) are parameters related to the rate of change in body weight and average body weight in the maintenance energy gap estimation, respectively.

| **Socioeconomic status** | $\overline{F}_0$ | $\phi_1$ | $\phi_2$ |
|---|---|---|---|
| *Low* | 17.8 | 8942.2 | 22.9 |
| *Middle* | 20.7 | 9285.2 | 22.0 |
| *High* | 21.9 | 9410.4 | 21.7 |

According to our estimations, the average energy intake in the Mexican adults has increased by SES during the last 18 years, leading to positive MEGs (Fig. B). The low SES had the highest changes in energy intake between 2000 and 2018 (134.6 kcal/day), followed by the middle SES (105.9 kcal/day) and the high SES (73.3 kcal/day). Each specific MEG by ENSANUT wave from $T_0 = 2000$ to $T_n = 2018$ are presented in Table D. In the next section, we detailed the process to project the maintenance energy gaps to 2030 and 2040.

**Fig B:** Average intake changes (kcal) between $T_0 = 2000$ and $T_n = 2006, 2012, 2016$ or 2018 by socioeconomic status. According to our estimations, there were increases in energy intake between 2000 and 2018 by socioeconomic status (SES): 134.6, 105.9 and 73.3 kcal/d for the low, middle and high SES respectively.

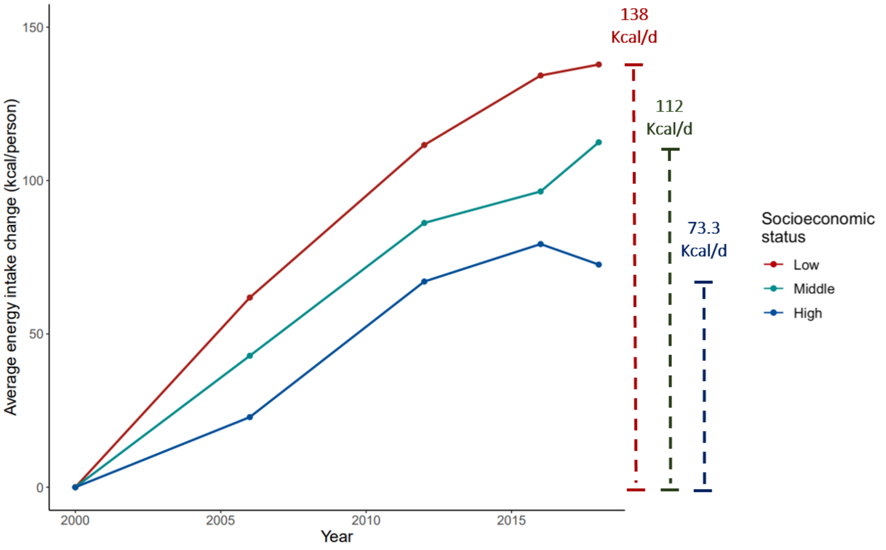


**Table D:** Energy gaps in Mexico between $T_0 = 2000$ and $T_n = 2006, 2012, 2016$ or 2018 by socioeconomic status.

| **Socioeconomic status** | 2006 | 2012 | 2016 | 2018 |
|---|---|---|---|---|
| *Low* | 62 | 112 | 134 | 138 |
| *Middle* | 43 | 86 | 96 | 112 |
| *High* | 23 | 67 | 79 | 73 |

## 2.2 Projecting Maintenance Energy Gaps over time

We projected MEGs by 2030 and 2040 using a linear fit with gradual reductions on the slope, as in the Nordpred model. The Nordpred is a mathematical

model that was previously used to predict trends in cancer incidence [10, 11] and is based on an exponential growing function with a special property: the exponential slope is gradually reduced by 5-year periods. For the first 5-year period, no reduction is considered. Then, the original slope is reduced 25% and 50% for the second and third 5-year period, respectively. For the subsequent years, a reduction of 75% is kept constant. Those reductions are made assuming that the increasing trend would flatten. For our analyses, we considered linear trends for the MEGs by SES groups:

$$\widehat{MEG}_k(T) = \beta_{0k} + \beta_{1k} \times t; \tag{6}$$

where $\widehat{MEG}_k(T)$ is the maintenance energy gap estimated at time $T$ for the SES group $k$, and $\beta_{0k}$ and $\beta_{1k}$ the estimated regression coefficients (Table E). We applied reductions to the slope of the linear fits ($\beta_{1k}$) from the baseline year 2018, according to the Nordpred reductions. A graphical comparison between the linear model and the Nordpred-based fit is shown in Figure C.

**Table E:** Linear model estimated for projecting maintenance energy gaps by socioeconomic status.

| **Socioeconomic status** | **Variable** | $\widehat{\beta}$ | $\widehat{se}(\widehat{\beta})$ | *p-value* | **Adjusted** $R$ *squared* ($R^2$) |
|---|---|---|---|---|---|
| *Low* | Intercept | -15,554.4 | 1,417.8 | 0.002 | 0.97 |
| | Year | 7.8 | 0.7 | 0.002 | |
| *Middle* | Intercept | -12,260.8 | 892.2 | < 0.001 | 0.98 |
| | Year | 6.1 | 0.4 | < 0.001 | |
| *High* | Intercept | -9145.2 | 1,354.3 | 0.006 | 0.92 |
| | Year | 4.6 | 0.6 | 0.006 | |

**Fig C:** Average changes in energy intake from 2000, projected by socioeconomic status using a linear fit and a Nordpred-based fit. The Nordpred-based fit starts with a linear trend (2000-2022), then gradual reductions are applied to its slope every 5 years. The linear slope of the Nordpred-based fit is reduced by 25% during 2023-2027, 50% during 2028-2032, and 75% in the subsequent years. The dotted gray line indicates the year 2018, which corresponds to the last database considered in the analysis (National Health and Nutrition Survey 2018). The solid points (●) are estimated maintenance energy gaps from 2000.

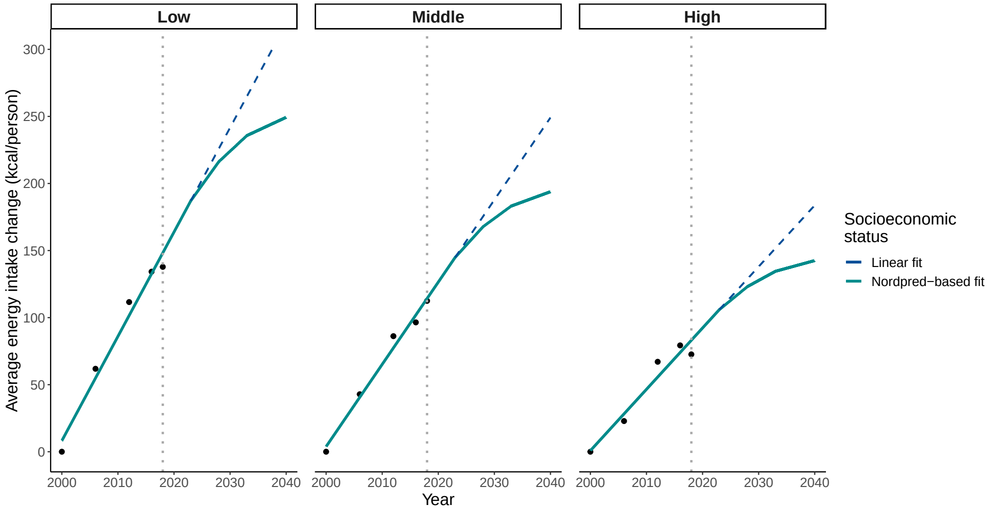


### 2.2.1 Maintenance energy gaps from the baseline year

We translated the MEG projections from $T_0$=2000 to $T_0^{'}$=2018 to match the year of the baseline sample (ENSANUT 2018):

$$\widehat{MEG}_k^{2018}(T) = \widehat{MEG}_k(T) - \widehat{MEG}_k(2018), \tag{7}$$

where $\widehat{MEG}_k^{2018}(T)$ is the projected MEG between $T_0^{'}$=2018 and time $T$ for the SES group $k$. The translated MEGs by SES are presented in Figure D. With the translation, we obtained MEGs with an initial time of $T_0^{'}$=2018, which was needed to simulate body weights in the next step.

**Fig D:** Average changes in energy intake projected from 2018 in Mexico by socioeconomic status using a Nordpred-based fit.

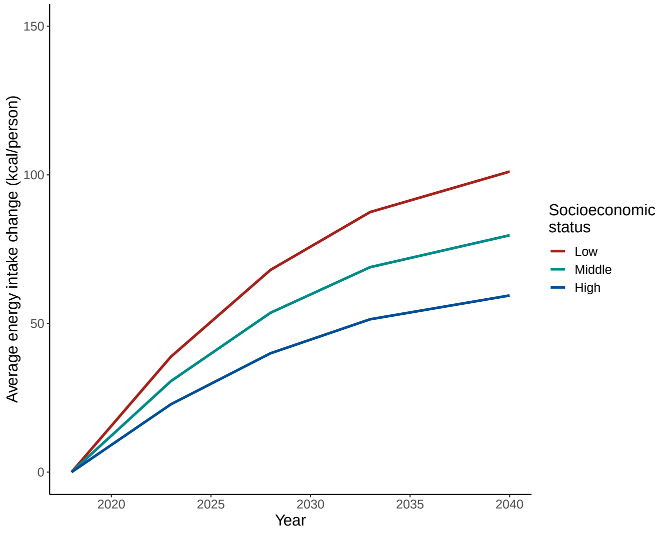


## 2.3 Simulating body weight using microsimulation

We simulated body weight using the microsimulation weight change model for adults proposed by Hall and colleagues [5]. To initialize the model, we used the individuals in the baseline sample considering their sex, age, weight, and height. The microsimulation model was completely programmed in the *bw* package [12] in $R$, and it was conducted using the projected MEGs detailed in the previous section ($\widehat{MEG}_k^{2018}$). First, we assigned a trend of MEGs to each individual $j$ in the baseline sample, according to its SES group $k$:

$$\overrightarrow{MEG}_j(t) = \left(\widehat{MEG}_k^{2018}(0),\ \widehat{MEG}_k^{2018}(1),\ \dots,\ \widehat{MEG}_k^{2018}(v),\ \dots,\ \widehat{MEG}_k^{2018}(t)\right); \tag{8}$$

where $v$ stands for the $v$-*th* day after the baseline year and $t$ is the number of days to simulate; for simulating by 2040, $t = (2040 - 2018) \times 365$. Body weight was simulated for each individual $j$ in the sample as

$$BW_j(t) = BW_j^{\text{model}}\big(t + age_j;\ \text{Sex}_j, \text{Height}_j(0), \text{BW}_j(0), \overrightarrow{PAL}_j(t) = \overrightarrow{1.5}, \overrightarrow{\Delta Na}_j(t) = \overrightarrow{0}, EIchange = \overrightarrow{MEG}_j(t)\big) \quad (9)$$

where $\text{Height}_j(0)$ and $\text{BW}_j(0)$ are the initial height and body weight, respectively. $\overrightarrow{PAL}_k(t)$ is the physical activity level by day assumed to be constant over time (sedentary level = 1.5), and $\overrightarrow{\Delta Na}_k(t)$ the daily changes in sodium, accounting for changes in extracellular fluid. $EIchange$ represents the daily changes in energy intake from baseline for the individual. In our case, the baseline is $T_0^{'} = 2018$ and $EIchange = \overrightarrow{MEG}_j(t)$, indicating increasing trends in consumption from baseline by SES under a business as usual scenario (Fig D).

## 2.4 Obesity prevalence considering sex and age distribution for 2030 and 2040

As we are predicting obesity over a long period of time, age and sex could bias our estimation. We used a sample balancing method "raking" to account for the changes expected in the sex and age distribution of the population from 2018 to 2030 and 2040. Raking is a statistical method that adjusts a set of data so that its marginal totals (not adjusted) match control totals (adjusted) [13]. With this method, we constructed two sets of sampling weights to match the age and sex distribution projected for 2030 and 2040.
The total population distribution by sex and age according to the National Population Council of Mexico for 2030 and 2040 is presented in Table F [14]. All of our results for 2030 and 2040 were estimated using the adjusted weights. Raking was performed using the "survey" package on R Statistical Software [2–4].

```
For 2030,

calibration.raking.2030 <- calibrate(
Design = Svy.design.2018,
formula = ~ sex_age.group.indicator.variable.2018,
population = ~ Expected.pop.by.sex.age.group.2030,
calfun = ''raking'')

ENSANUT2018$weights.2030 <- weights(calibration.raking.2030)
```

```
For 2040,

calibration.raking.2040 <- calibrate(
Design = Svy.design.2018,
formula = ~ sex_age.group.indicator.variable.2018,
population = ~ Expected.pop.by.sex.age.group.2040,
calfun = ``raking'')

ENSANUT2018$weights.2040 <- weights(calibration.raking.2040)
```

**Table F:** Mexican adult population stratified by sex and age group for 2018 and the expected population for 2030 and 2040.

| **Sex and age group** | **Total population** | | |
|---|---|---|---|
| | **2018*** | **2030**** | **2040**** |
| Female 20-29 | 8,471,630 | 10,630,763 | 10,338,617 |
| Female 30-39 | 7,905,467 | 10,412,825 | 10,359,876 |
| Female 40-49 | 9,340,607 | 9,541,932 | 10,162,244 |
| Female 50-59 | 7,506,259 | 8,195,137 | 9,165,431 |
| Female 60-69 | 5,734,956 | 6,052,624 | 7,508,501 |
| Female 70-79 | 2,985,834 | 3,484,976 | 4,937,064 |
| Female 80+ | 1,721,427 | 1,663,811 | 2,609,797 |
| Male 20-29 | 7,334,823 | 10,784,666 | 10,513,016 |
| Male 30-39 | 5,908,620 | 10,260,888 | 10,296,796 |
| Male 40-49 | 6,748,167 | 8,704,200 | 9,860,966 |
| Male 50-59 | 5,618,698 | 7,105,397 | 8,170,824 |
| Male 60-69 | 3,985,169 | 5,250,936 | 6,222,225 |
| Male 70-79 | 2,525,323 | 2,845,507 | 3,948,738 |
| Male 80+ | 1,250,405 | 1,202,084 | 1,847,999 |
| Overall | 77,037,385 | 96,135,746 | 105,942,094 |

* Population estimated for 2018 based on the National Health and Nutrition Survey

** Projected population obtained from the National Population Council of Mexico

## 2.5 Cross-model validation

For comparing our results, we projected MEGs using a root square or a Gompertz fit as alternative projections to the Nordpred-based fit. We also considered a multinomial regression model previously used to project obesity trends in the US at a population level [15]. Those alternative fits and the multinomial model are detailed in the next subsections.

### 2.5.1 Root square fit

We adjusted a root squared curve for projecting MEGs by SES groups using the following equation:

$$MEG_k(T) = \sqrt{\beta_{0k} + \beta_{1k} \times T} - a_k; \tag{10}$$

where $k$ represents the SES group. We estimated $\beta_{0k}$ and $\beta_{1k}$ using a linear regression as follows

$$\left(MEG_k(T) + a_k\right)^2 = \beta_{0k} + \beta_{1k} \times T. \tag{11}$$

$a_k$ was chosen to ensure that $\widehat{\beta}_{0k} + \widehat{\beta}_{1k} \times T \geq 0$ for all $T \geq 2000$, which was a required property to apply the root square on that range of values. It was sufficient to verify that property only for $T_0 = 2000$ since $f(t) = \widehat{\beta}_{0k} + \widehat{\beta}_{1k} \times T$ is an increasing function of $T$. $a_k$ was also chosen such that the energy balance assumption at $T_0$ approximately held; i.e., $\widehat{MEG}_k(T_0) \approx 0$. Table G shows the estimation of $\widehat{MEG}_k(T_0)$ for different values of $a_k$, and Table H presents the coefficients estimated for projecting MEGs.

**Table G:** Maintenance energy gap estimated by a root square fit for the initial time ($T_0 = 2000$) varying one of its parameters ($a_k$). For some values of $a_k$, the maintenance energy gap does not exist (undefined) because the root square function is only defined over non-negative values ($\geq 0$).

| **Socioeconomic status** | $a_k$ | $\widehat{\beta}_{0k} + \widehat{\beta}_{1k} \times T_0$ | $\widehat{MEG}_k(T_0) = \sqrt{\beta_{0k} + \beta_{1k} \times t} - a_k$ |
|---|---|---|---|
| *Low* | 0.0 | -970.5 | Undefined |
| | 50.0 | 2,191.4 | -3.2 |
| | 68.8 | 4,732.1 | $-9.5 \times 10^{-3}$ |
| | 68.9* | 4,747.5 | $2.4 \times 10^{-4}$ |
| *Middle* | 0.0 | -1,187.4 | Undefined |
| | 100.0 | 9792.4 | $-1.0$ |
| | 127.1 | 16,153.5 | $-3.5 \times 10^{-3}$ |
| | 127.2* | 16,179.7 | $4.6 \times 10^{-4}$ |
| *High* | 0.0 | -753.5 | Undefined |
| | 300.0 | 89,994.16 | $9.7 \times 10^{-3}$ |
| | 303.5 | 92,112.0 | $-3.6 \times 10^{-4}$ |
| | 303.6* | 92,172.9 | $-9.9 \times 10^{-5}$ |

*Parameter used for projecting maintenance energy gaps over time. This value was chosen such that $\widehat{MEG}_k(T_0)$ is defined and $\widehat{MEG}_k(T_0) \approx 0$ (energy balance assumption at $T_0 = 2000$).

**Table H:** Root square model estimated for projecting maintenance energy gaps by socioeconomic status.

| **Socioeconomic status** | $a_k$ | **Variable** | $\widehat{\beta}$ | $\widehat{se}(\widehat{\beta})$ | *p-value* | **Adjusted** $R$ *squared* $(R^2)$ |
|---|---|---|---|---|---|---|
| *Low* | 68.9 | Intercept | $-4.4 \times 10^6$ | $2.1 \times 10^5$ | $< 0.001$ | 0.99 |
| | | Year | $2.2 \times 10^3$ | $1.0 \times 10^2$ | $< 0.001$ | |
| *Middle* | 127.2 | Intercept | $-4.5 \times 10^6$ | $2.6 \times 10^5$ | $< 0.001$ | 0.99 |
| | | Year | $2.2 \times 10^3$ | $1.3 \times 10^2$ | $< 0.001$ | |
| *High* | 303.6 | Intercept | $-6.2 \times 10^7$ | $9.5 \times 10^6$ | 0.007 | 0.92 |
| | | Year | $3.1 \times 10^3$ | $4.7 \times 10^2$ | 0.007 | |

### 2.5.2 Gompertz model

Gompertz model is a classic mathematical model previously used to describe growth data in different fields of study, such as biology, economics, and others [16–19]. Gompertz model belongs to the family of sigmoidal curves, which are known by having an "S"-shape and horizontal asymptotes that constrain their growth. For our analyses, we considered the Gompertz model to project MEGs by SES as follows: [20]

$$(MEG_k(T) + 1) = asympt_k \times exp\left\{-b_k \times c_k^{(T-T_0)}\right\}; \qquad (12)$$

where $k$ represents the *k-th* SES group, $asympt_k$ the asymptote of the curve, $b_k$ a parameter controlling the displacement on the $x$-axis, and $c_k$ the growth rate. For each SES group, we took the asymptote as the maximum value of the adjusted linear trend for the group, allowing growth for the Gompertz trend (Table I).

**Table I:** Maximum values estimated for the projections of energy changes from 2000 to 2040 in Mexico by socioeconomic status. These maximum values corresponded to the values reached in 2040 since the projections were estimated from increasing functions

| Socioeconomic status | Model | Maximum value estimated by the model (kcal/person) |
|---|---|---|
| Low | Linear | 319 |
| | Nordpred | 250 |
| | Root square | 236 |
| Middle | Linear | 249 |
| | Nordpred | 194 |
| | Root square | 199 |
| High | Linear | 184 |
| | Nordpred | 143 |
| | Root square | 163 |

The parameters $b_k$ were estimated at $T = T_0$ from equation (12):

$$b_k = log(asympt_k). \tag{13}$$

The parameters $c_k$ can be obtained from equations (12) and (13) for $T > T_0$ as follows

$$c_k = -(b_k)^{-1} \times exp\left\{(T - T_0)^{-1}\right\} \times log\left\{MEG_k(T) \times (asympt_k)^{-1}\right\} \tag{14}$$

We estimated $c_k$ for $T$ = 2006, 2012, 2016, 2018 (years of the ENSANUT) and took the $c_k$ value with the lowest error values by SES group. Since the Gompertz model was parameterized analytically, no uncertainty estimation was calculated from it. Instead, we took the standard deviations (SD) of the Nordpred-based projections to build uncertainty around the Gompertz projections. Compared to the root square fit, the Nordpred-based projections had greater values of SD, considering more uncertainty in the estimations. Initially, least-square approaches were used for the Gompertz models. However, those approaches limited the growth of the curve, estimating low values for the asymptotes. The parameters used for the Gompertz model are summarized in Table J.

**Table J:** Gompertz model estimated for projecting maintenance energy gaps by socioeconomic status.

| **Socioeconomic status** | Asymptote | $b_k$ | $c_k$ |
|---|---|---|---|
| *Low* | 319 | 5.8 | 0.9 |
| *Middle* | 249 | 5.5 | 0.9 |
| *High* | 184 | 5.2 | 0.9 |

### 2.5.3 Comparing projections of maintenance energy gaps

Figure E presents the projections of MEGs by SES according to the linear, Nordpred-based model, root square fit, and the Gompertz model. In all models, the low SES group is increasing faster than the other groups. The adjusted R-squared and the RMSE of the models indicated that they fitted well to the points (Table K). However, according to the root mean square error (RMSE), the Nordpred-based/linear and the root square fit adjusted best to the points (RMSE $\leq$ 8.1), compared to the Gompertz model (RMSE $\leq$ 26.6)

**Fig E:** Projections of average changes in energy intake for 2000-2040 in Mexico by socioeconomic status, using four different approaches: a linear fit, a Nordpred-based fit, a Root square fit, and the Gompertz model. The solid points (•) are the estimated maintenance energy gaps and represent increases in energy intake from $T_0 = 2000$. The dashed line indicates the year of the last survey wave considered (2018).

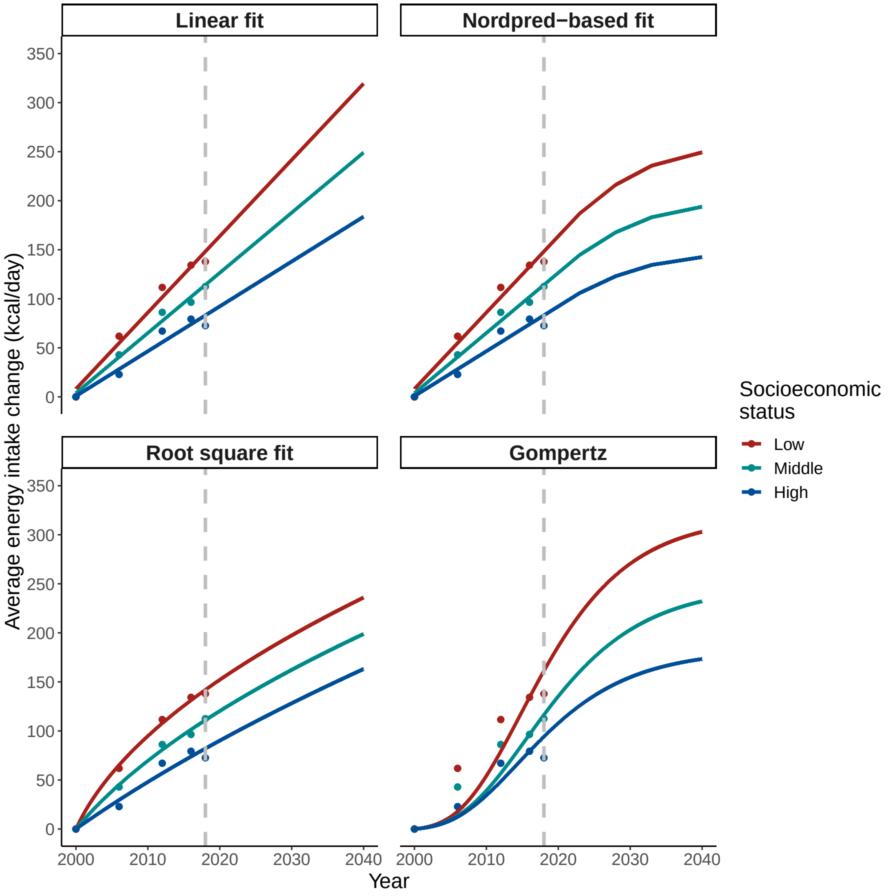

**Table K:** Goodness-of-fit measures estimated for the energy-projection models Nordpred-based, linear, root square, and Gompertz adjusted by socioeconomic status

| Socioeconomic status | Model | Adjusted R squared | RMSE[a] |
|---|---|---|---|
| Low | Nordpred-based/linear | 0.97 | 8.1 |
| | Root square | 0.99 | 3.2 |
| | Gompertz[b] | - | 26.6 |
| Middle | Nordpred-based/linear | 0.98 | 5.1 |
| | Root square | 0.99 | 3.5 |
| | Gompertz | - | 18.6 |
| High | Nordpred-based/linear | 0.91 | 7.7 |
| | Root square | 0.92 | 7.4 |
| | Gompertz | - | 24.4 |

[a]RMSE = Root mean square error
[b] Gompertz fit was estimated analytically. No adjusted R squared or similar measure was estimated

### 2.5.4 Multinomial regression model

We simulated obesity prevalence at a population level using a multinomial regression (Mlogit) model proposed by Ward et al. [15]. This model simulated the prevalence by body mass index (BMI) category $i = 1$ to 3 (normal weight, overweight, and obesity) at year $t$ (2030 and 2040) as:

$$Prev(i,t) = \frac{\sigma_i(t)}{\sum_{m=1}^{3} \sigma_m(t)};$$

where

$$\sigma_i(t) = \frac{exp\,(\beta_{0i} + \beta_{1i} \cdot t)}{1 + exp\,(\beta_{0i} + \beta_{1i} \cdot t)}.$$

Mlogit model ensures that the sum of the three categories is equal to 100% for 2030 and 2040.

### 2.5.5 Comparing results for obesity prevalence

Fig. F shows the projected obesity prevalence by 2030 and 2040 for the Nordpred-based, root square, Gompertz, and the multinomial approach, in-

cluding intervals accounting for the uncertainty of the projected MEGs (section 5). The uncertainty intervals (UI) of the Nordpred-based fit include the prevalence of the other projections for 2030 and 2040.

**Fig F:** Comparison of obesity prevalence projections by 2030 and 2040 for Mexican adults under different models.

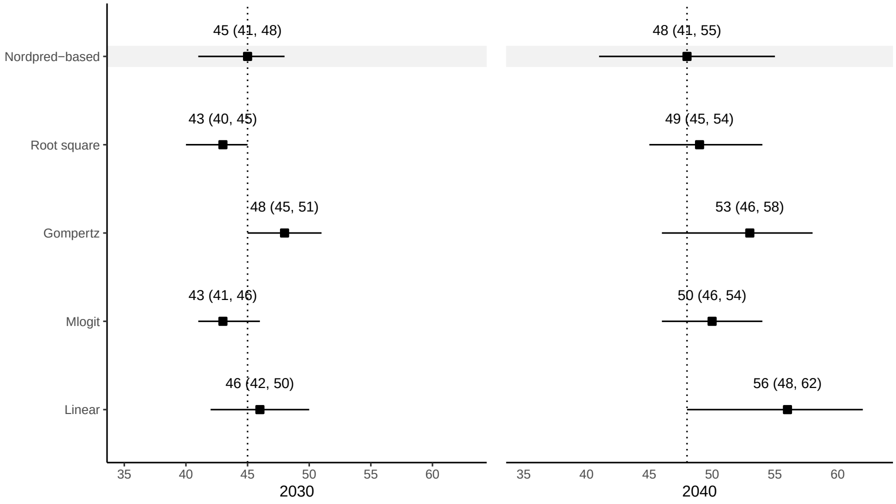


### 2.5.6 Comparison with other estimations in Mexico

We compared our prevalence projections by 2030 with the ones estimated by Sánchez-Romero using a linear model. [21] Table S6 shows the estimated prevalence by age and sex. There are small differences between the estimates and most confidence intervals overlap.

**Table L:** Comparing projections of obesity prevalence for 2030 by sex and age group.

| Age group | Obesity prevalence by 2030 % (UI[a]) — Male: Microsimulation approach | Male: Sánchez-Romero (2017) | Female: Microsimulation approach | Female: Sánchez-Romero (2017) |
|---|---|---|---|---|
| 20-24 | 22.3 (20.8 ,24.5) | 29.4 (24.3, 34.5) | 30.8 (27.2, 33.9) | 29.8 (22.8, 36.8) |
| 25-29 | 36.9 (33.4, 40.1) | 38.9 (37.2, 40.6) | 41.3 (36.1, 42.7) | 40.0 (32.4, 47.6) |
| 30-34 | 38.1 (34.9, 41.0) | 42.5 (32.7, 52.2) | 44.2 (40.5, 50.2) | 47.6 (41.3, 53.9) |
| 35-39 | 49.5 (42.9, 52.4) | 52.0 (43.6, 60.4) | 52.1 (48.8, 57.7) | 57.4 (53.5, 61.3) |
| 40-44 | 48.7 (44.8, 51.6) | 54.4 (45.3, 63.5) | 60.8 (57.2, 63.8) | 50.3 (46.2, 54.5) |
| 45-49 | 46.2 (42.8, 50.5) | 35.9 (10.8, 61.1) | 59.6 (54.3, 64) | 61.0 (52.6, 69.3) |
| 50-54 | 48.3 (43.5, 50.3) | 34.5 (22.0, 47.0) | 59.1 (55.5, 63) | 59.9 (42.8, 77.1) |
| 55-59 | 34.2 (30.7, 38.3) | 32.9 (13.4, 52.4) | 56.0 (51.2, 61.9) | 57.7 (46.0, 69.5) |
| 60-64 | 44.5 (40.6, 46.6) | 28.3 (18.7, 38.0) | 60.6 (58.4, 64.7) | 56.5 (37.5, 75.5) |
| 65-69 | 31.7 (29.3, 38.1) | 17.0 (5.0, 29.0) | 53.2 (50.0, 57.2) | 54.5 (48.3, 60.6) |
| 70-74 | 35.1 (28.6, 41.8) | 27.2 (10.8, 43.7) | 48.6 (45.1, 55.6) | 54.9 (40.7, 69.2) |
| 75-79 | 25.1 (23.5, 35.7) | 20.8 (0.0[b], 49.6) | 42.3 (37.8, 45.1) | 36.0 (25.2, 46.7) |
| $\geq 80$ | 24.2 (19.7, 25.7) | 8.2 (3.4, 12.9) | 34.0 (25.8, 36.5) | 23.5 (3.5, 43.4) |
| *All ages* | 38.2 (34.7, 41.5) | 37.8 (31.1, 42.5) | 49.6 (45.3, 53.2) | 52.1 (48.6, 55.7) |

[a]UI = Uncertainty interval

[b]Estimate < 0

# 3 Defining obesity goals for 2030 and 2040

## 3.1 Piloting

The questionnaire was designed and piloted by us. For that, we submitted it to two nutrition experts and asked them to read all questions, the ranges of responses and comment on different aspects such as comprehension and appropriateness. Redaction changes were made in response to their comments. Once we had a final version of the questionnaire, we sent the questionnaire by email to nine obesity and nutrition experts in Mexico. We obtained response by 8 out ot 9 experts. The final questionnaire in Spanish is available online [22].

# 4 Caloric reductions to achieve the obesity goals

**Fig G:** Average energy intake changes projected from 2018 for the low socioeconomic status using the Nordpred-based fit. The solid line represents the scenario under no intervention, while the dashed line represents the intervention scenario. The intervention scenario results from applying a constant reduction of MEG = 101 kcal/person/day to the scenario without intervention. MEG = 101 kcal/person corresponds to the increase in intake from 2018 to 2040 estimated for the low socioeconomic status in the scenario without intervention.

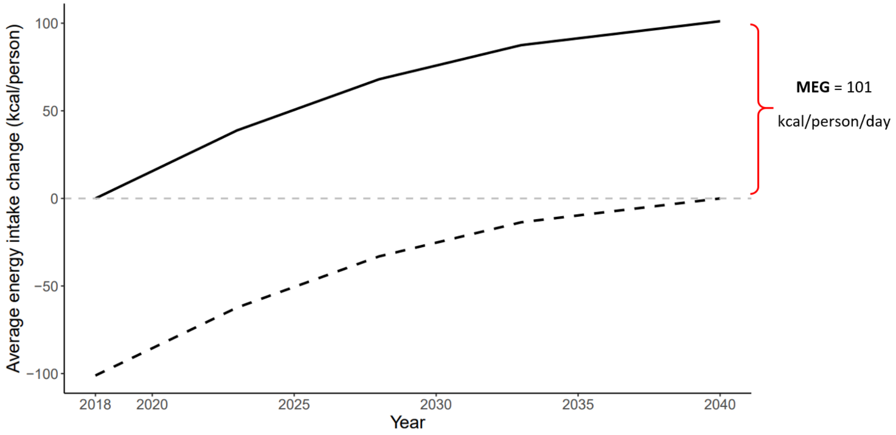


**Table M:** Energy intake by socioeconomic status by 2018 in Mexico. These intakes were estimated assuming that people in 2018 were in energy equilibrium (Energy intake = Energy expenditure) and had a sedentary physical activity level (PAL = 1.5).

| | **Energy intake; kcal/person (UI)**[a] | | | |
|---|---|---|---|---|
| | | **Socioeconomic status** | | |
| Scenario | Total population | Low | Middle | High |
| **Energy intake** | 2108 (2097, 2118) | 2041 (2025, 2056) | 2130 (2114, 2146) | 2143 (2122, 2165) |

**UI = Uncertainty interval**

**Table N:** Obesity prevalence goals in 2030 and 2040 by socioeconomic status and caloric gaps needed to achieve them. The gaps are presented as relative values (%) of the energy intakes estimated by 2018.

| | | **Caloric gaps to achieve the goals; % of intake estimated in 2018 (UI[a])** | | | |
|---|---|---|---|---|---|
| | | | **Socioeconomic status** | | |
| Scenario | Obesity goal | Total population | Low | Middle | High |
| **2030** | | | | | |
| Ideal (OECD[b]) | 19 | -9 (-10, -8) | -10 (-11, -9) | -9 (-10, -8) | -8 (-10, -7) |
| Intermediate | 33 | -4 (-4, -2) | -4 (-5, -3) | -4 (-4, -2) | -3 (-4, -1) |
| Plausible | 38 | -2 (-3, -1) | -3 (-4, -2) | -2 (-3, -1) | -1 (-2, 0 [c]) |
| **2040** | | | | | |
| Ideal (OECD) | 19 | -10 (-12, -8) | -11 (-13, -9) | -10 (-12, -8) | -9 (-12, -6) |
| Intermediate | 33 | -6 (-8, -4) | -7 (-9, -5) | -6 (-8, -4) | -5 (-8, -2) |
| Plausible | 38 | -4 (-5, -1) | -5 (-6, -3) | -4 (-5, -2) | -3 (-5, 0 [c]) |

[a]**UI = Uncertainty interval**

[b]**Average obesity prevalence from the countries included in the Organization for Economic Co-operation and Development in 2017**

[c] **Estimate** $> 0$

# 5 Uncertainty analysis

**Fig H:** Projections of average changes in energy intake for 2000-2040 in Mexico by socioeconomic status, using four different approaches: a linear fit, a Nordpred-based fit, a Root square fit, and the Gompertz model. The solid curve represents the maintenance energy gaps predicted. The black curves indicate the uncertainty intervals estimated using simulation.

**Linear**

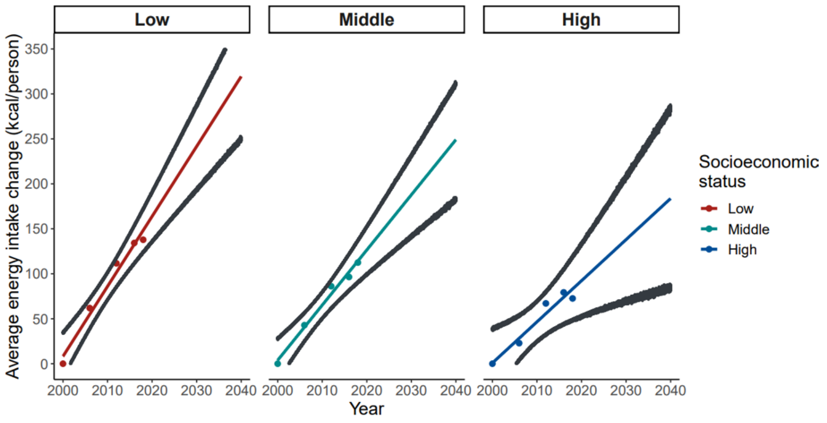

**Nordpred-based fit**

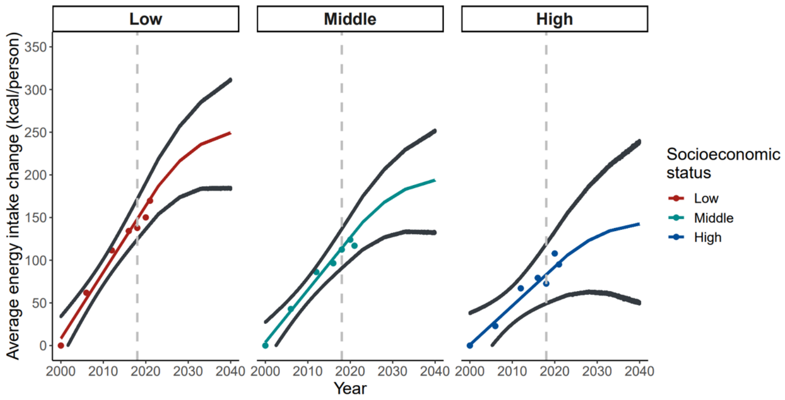


**Root square fit**

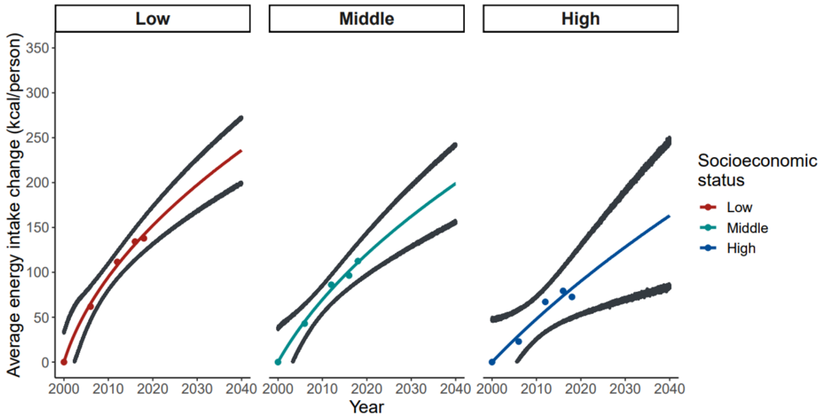

## Gompertz

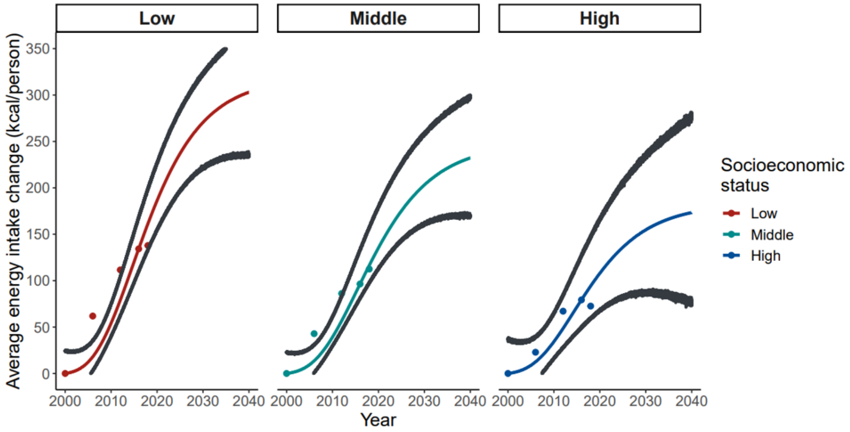

Low
Middle
High
Average energy intake change (kcal/person)
350
300
250
200
150
100
50
0
2000
2010
2020
2030
2040
Year
Socioeconomic status
Low
Middle
High

# References


[1] Kolenikov S, Angeles G. The Use of Discrete Data in PCA: Theory, Simulations, and Applications to Socioeconomic Indices. Chapel Hill, N.C.: Carolina Population Center MEASURE. 2004.

[2] Lumley T. survey: analysis of complex survey samples; 2020.

[3] Lumley T. Complex Surveys: A Guide to Analysis Using R: A Guide to Analysis Using R. John Wiley and Sons; 2010.

[4] R Core Team. R: A Language and Environment for Statistical Computing. Vienna, Austria: R Foundation for Statistical Computing; 2021. Available from: https://www.R-project.org/.

[5] Hall KD, Sacks G, Chandramohan D, Chow CC, Wang YC, Gortmaker SL, et al. Quantification of the effect of energy imbalance on bodyweight. The Lancet. 2011 8;378(9793):826-37.

[6] Hall KD, Guo J, Dore M, Chow CC. The Progressive Increase of Food Waste in America and Its Environmental Impact. PLOS ONE. 2009 11;4(11):e7940. Available from: https://doi.org/10.1371/journal.pone.0007940.

[7] Nelson KM, Weinsier RL, Long CL, Schutz Y. Prediction of resting energy expenditure from fat-free mass and fat mass. The American Journal of Clinical Nutrition. 1992 11;56(5):848-56.

[8] Forbes GB. Lean Body Mass-Body Fat Interrelationships in Humans. Nutrition Reviews. 2009 4;45(10):225-31.

[9] Jackson A, Stanforth P, Gagnon J, Rankinen T, Leon A, Rao D, et al. The effect of sex, age and race on estimating percentage body fat from body mass index: The Heritage Family Study. International Journal of Obesity. 2002 6;26(6):789-96.

[10] Møller B, Fekjaer H, Hakulinen T, Tryggvadóttir L, Storm HH, Talbäck M, et al. Prediction of cancer incidence in the Nordic countries up to the year 2020. European journal of cancer prevention : the official journal of the European Cancer Prevention Organisation (ECP). 2002 6;11 Suppl 1:1-96.

[11] Møller B, Fekjaer H, Hakulinen T, Sigvaldason H, Storm HH, Talbäck M, et al. Prediction of cancer incidence in the Nordic countries: empirical comparison of different approaches. Statistics in Medicine. 2003 9;22(17):2751-66.

[12] Camacho-García-Formentí D, Zepeda-Tello R. bw; 2018.

[13] Battaglia MP, Hoaglin DC, Frankel MR. Practical Considerations in Raking Survey Data. Survey Practice. 2009 6;2(5):1-10.

[14] Consejo Nacional de Poblacion. Proyecciones de los hogares en México y las Entidades Federativas, 2016–2050 In: México en cifras. 2014. Available from: `https://datos.gob.mx/busca/dataset/proyecciones-de-la-poblacion-de-mexico-y-de-las-entidades-federativas-2016-2050`.

[15] Ward ZJ, Bleich SN, Cradock AL, Barrett JL, Giles CM, Flax C, et al. Projected U.S. State-Level Prevalence of Adult Obesity and Severe Obesity. New England Journal of Medicine. 2019 12;381(25):2440-50.

[16] Hernandez-Llamas A, Ratkowsky DA. Growth of fishes, crustaceans and molluscs: estimation of the von Bertalanffy, Logistic, Gompertz and Richards curves and a new growth model. Marine Ecology Progress Series. 2004;282:237-44. Available from: `https://www.int-res.com/abstracts/meps/v282/p237-244/`.

[17] Pradhan T, Chaudhuri KS. Bioeconomic Modelling of a Single Species Fishery with Gompertz Law of Growth. Journal of Biological Systems. 1998 12;06(04):393-409.

[18] Moura NJ, Ribeiro MB. Evidence for the Gompertz curve in the income distribution of Brazil 1978–2005. The European Physical Journal B. 2009 1;67(1):101-20.

[19] Lloyd HH. Estimation of tumor cell kill from Gompertz growth curves. Cancer chemotherapy reports;59(2 Pt 1):267-77.

[20] Panik MJ. Growth Curve Modeling. Hoboken, NJ: John Wiley & Sons, Inc; 2014.

[21] Sánchez Romero LM. Impact of future obesity trends in the Mexican population: Development of a computer simulation model. PhD thesis, University College London; 2017. Available from: `https://discovery.ucl.ac.uk/id/eprint/1559914/1/Sanchez-Romero_ID_PhD_Thesis_FINAL.pdf`.

[22] Basto-Abreu A, González-Morales R, Canto-Osorio F, Torres-Alvarez R. Cuestionario a expertos en obesidad y nutrición;. Available from: https://docs.google.com/forms/d/e/1FAIpQLSdf6BWILVMPVcYW5a2GS6gyxdkbIuFx2IU0XUumwMl3WC7Khw/viewform?vc=0&c=0&w=1&flr=0.
